# Tuning Mechanical Properties, Swelling, and Enzymatic Degradation of Chitosan Cryogels Using Diglycidyl Ethers of Glycols with Different Chain Length as Cross-Linkers

**DOI:** 10.3390/gels10070483

**Published:** 2024-07-21

**Authors:** Yuliya Privar, Anna Skatova, Mariya Maiorova, Alexey Golikov, Andrey Boroda, Svetlana Bratskaya

**Affiliations:** 1 Institute of Chemistry Far Eastern Branch of the Russian Academy of Sciences, 159, Prosp. 100-Letiya Vladivostoka, 690022 Vladivostok, Russia; 2 A.V. Zhirmunsky National Scientific Center of Marine Biology, Far Eastern Branch of Russian Academy of Sciences, 17, Palchevskogo Street, 690041 Vladivostok, Russia

**Keywords:** chitosan, cryogel, cross-linking, mechanical properties, cytotoxicity, stiffness, β-glucanase: FT-IR spectroscopy

## Abstract

Cross-linking chitosan at room and subzero temperature using a series of diglycidyl ethers of glycols (DEs)—ethylene glycol (EGDE), 1,4-butanediol (BDDE), and poly(ethylene glycol) (PEGDE) has been investigated to demonstrate that DEs can be a more powerful alternative to glutaraldehyde (GA) for fabrication of biocompatible chitosan cryogels with tunable properties. Gelation of chitosan with DEs was significantly slower than with GA, allowing formation of cryogels with larger pores and higher permeability, more suitable for flow-through applications and cell culturing. Increased hydration of the cross-links with increased DE chain length weakened intermolecular hydrogen bonding in chitosan and improved cryogel elasticity. At high cross-linking ratios (DE:chitosan 1:4), the toughness and compressive strength of the cryogels decreased in the order EGDE > BDDE > PEGDE. By varying the DE chain length and concentration, permeable chitosan cryogels with elasticity moduli from 10.4 ± 0.8 to 41 ± 3 kPa, toughness from 2.68 ± 0.5 to 8.3 ± 0.1 kJ/m^3^, and compressive strength at 75% strain from 11 ± 2 to 33 ± 4 kPa were fabricated. Susceptibility of cryogels to enzymatic hydrolysis was identified as the parameter most sensitive to cross-linking conditions. Weight loss of cryogels increased with increased DE chain length, and degradation rate of PEGDE-cross-linked chitosan decreased 612-fold, when the cross-linker concentration increased 20-fold.

## 1. Introduction

Biopolymer-based hydrogels and cryogels have been attracting increasing attention for different applications in biomedicine [1,2,3,4], wearable electronics [5], pollutants adsorption and detection [6,7,8], catalysis [9] etc. In comparison with hydrogels, cryogels benefit from an open porous structure, allowing efficient liquid flow or cell infiltration, higher mechanical strength and elasticity, and fast response to the external stimuli [10,11]. All these characteristics depend not only on the polymer nature but also on cryogelation conditions, cross-linking type, and density, which must be tuned to mimic native mechanical properties of the specific tissue [12,13,14], to control the rate of the scaffold resorption [14] or to optimize hydrodynamic properties of the flow-through filters and reactors [8].

A wide range of cross-linkers are applicable for polysaccharides and proteins [1,10,15,16], but glutaraldehyde (GA) remains one of the most used for biopolymers in general [15] and definitely the most used for chitosan [17,18,19,20]. In fact, the popularity of GA is based more on its low cost and well-established practice rather than on best performance. GA was shown to be cytotoxic at high concentrations, which are required to produce materials with high mechanical strength [17,18]. The removal of unreacted GA and toxic sodium borohydride used to reduce the formed Schiff’s base can be time-consuming and expensive [14]. Fast gelation of chitosan even at low GA concentrations [21,22] interferes with crystal growth in freezing solution and, therefore, limits the possibilities to control the pore size of chitosan cryogels [23]. Further, GA does not prevent strong interchain hydrogen bonding responsible for brittleness of polysaccharide materials. For example, the elasticity enhancement reached through mixing chitosan with gelatin was lost with the increase in the chitosan content in GA-cross-linked cryogels [24].

One of the simplest and the most efficient ways to increase the elasticity of hydrogels consists of using long-chain cross-linkers [25], which can be selected from a wide range of available diglycidyl ethers of glycols (DEs). DEs are FDA-approved reagents for biomedical applications including cross-linking commercially available hyaluronic acid hydrogels [26,27] and fixation of bioprosthetic tissue valves [28]. Cross-linking of hyaluronic acid with DEs proceeds via the epoxide ring opening reaction with the hydroxyl group in strong alkaline media [10,27]. These conditions are not applicable for fabrication of chitosan hydrogels and cryogels due to the polymer precipitation, but can be used for chitosan derivatives soluble in neutral and alkaline media [29,30] or pre-neutralized chitosan films [31].

In acidic media, the PEG-based cross-links were formed via chitosan reaction with PEG-dialdehyde in acetic acid/methanol medium followed by the reduction of the Schiff’s base with sodium cyanoborohydride [32], but the products obtained required purification through dialysis due to toxicity of both reagents. There are just a few examples of chitosan cross-linking with DE in acidic media, which are of interest for fabrication of chitosan cryogels. Kiuchi et al. reported chitosan cross-linking with PEGDE in 0.4% acetic acid solution at 80 °C during 24 h [33] and showed that the higher was the PEG content in the hydrogel film, the lower was the tensile strength and the higher was the elongation. We have shown that the reaction between chitosan and DEs proceeds much more efficiently in hydrochloric acid solutions that enabled us to fabricate mechanically stable chitosan cryogels at −10 °C [34], which were later used as scaffolds for 3D cell cultivation [35,36,37] and flow-through filters for dye adsorption [38].

Although the chitosan deacetylation degree and crystallinity are key parameters affecting the rate of its enzymatic degradation [39], enzymes recognize a certain sequence of monomer units in chitosan molecules [40], so that chemical modification at the amino group can change the monomer distribution pattern and reduce the number of structural fragments accessible for the enzymatic attack [40], leading to the lower degradation rate [31,41]. Taking the latter into account, one can expect that the mechanism of chitosan cross-linking can significantly affect the rate of enzymatic degradation.

Higher cross-linking density usually results in a reduced penetration of the enzymes into the chitosan network and slows degradation [42]; however, contradictory data were reported for the few available by now examples of DE-cross-linked chitosan and other polysaccharides. Despite the assumption about chitosan cross-linking via amino groups, all chitosan films neutralized with NaOH and cross-linked with DE of 1,4-butandiol (BDDE) in isopropanol at 45 °C were degraded with chitosanase within 24 h, demonstrating a minor difference between cross-linked and uncross-linked films [31]. For chitosan hydrogel films cross-linked with PEGDE in acetic acid medium, the increase in total PEG content resulted in a considerable increase in the degradation rate that was related to the high hydrophilicity of the cross-linked films [43], although cross-linking with DEs in acidic media is expected to reduce the number of accessible amino groups and, thus, the susceptibility to enzymatic hydrolysis [44]. In contrast, cross-linking of carboxymethylcellulose in alkaline media with PEGDE via hydroxyl groups decreased the enzymatic degradation rate, whereas correlation was found between degradability and swelling [45].

To sum up, one can conclude that DEs can be considered as more powerful alternative to GA for fabrication of chitosan hydrogels and cryogels, but their application is significantly limited by slow progress in chemistry of epoxide–amine reactions in acidic chitosan solutions and contradictory information on correlations between cross-linking conditions and materials properties. These facts have motivated us to undertake this systematic study and elucidate how the DE chain length and concentration affect porosity, swelling, mechanical properties, and enzymatic degradability of covalently cross-linked chitosan cryogels.

## 2. Results and Discussion

### 2.1. Gelation Time at Room Temperature 

To comply with principles of cryogel fabrication, it is mandatory that covalent cross-links are formed after the solvent crystallization. Only in this case ice crystals serve as templates for the macropores, whose parameters can be tuned through variation of cryogelation conditions. It is known that, at high concentrations of the cross-linker and the polymer, very rapid formation of primary cross-links interferes with ice crystals growth and results in heterogeneously cross-linked networks with zones of high and low local density of junctions [46]. For this reason, the cross-linking rate significantly affects the morphology of the cryogels.

Gelation of chitosan solutions in the presence of GA proceeds in a few minutes [21,22] that, in most cases, sets the upper limit of chitosan concentration for fabrication of cryogels to 2% [7,46]. In preliminary experiments we have observed that, in 3% chitosan solution at the GA concentration above GA:CH molar ratios 1:4, cross-linking reaction was so fast that hydrogels were formed before solvent crystallization, preventing generation of a macroporous structure.

**Figure 1:**
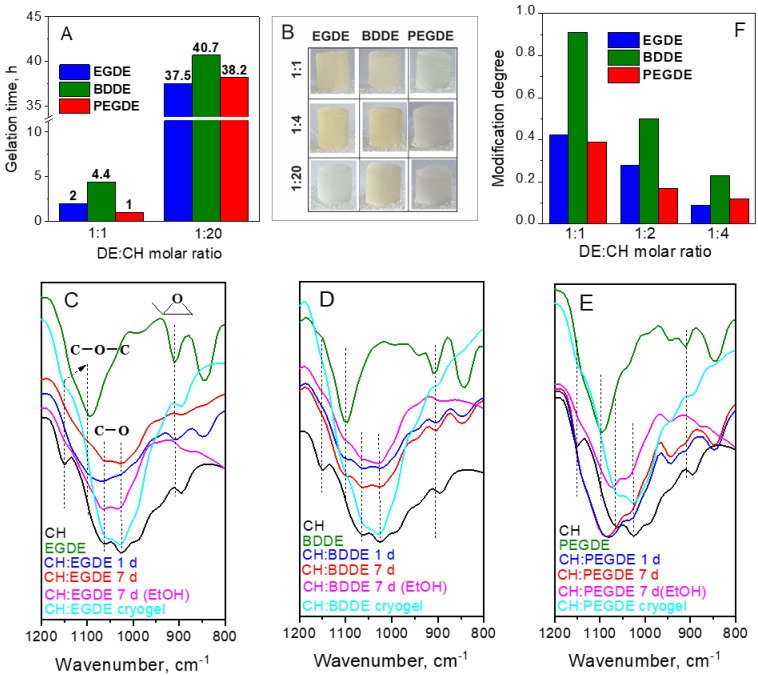
Dependence of gelation time in 3% chitosan (CH) solution, pH 5.5 on DE:CH molar ratio and DE chain length (**A**). Photo of CH cryogels obtained at different DE:CH molar ratios (**B**). FT-IR spectra of CH cryogels and hydrogels formed at 1st day (1 d) and 7th day (7 d) of gelation, before (no label) and after (EtOH) removal of unreacted reagents: cross-linking at equimolar ratio with EGDE (**C**), BDDE (**D**), and PEGDE (**E**). Dependence of the modification degree of chitosan cryogels with cross-linkers at different molar ratios DE:CH (**F**).

In comparison with GA-chitosan solutions, chitosan cross-linking with DEs proceeded much slower (Figure 1A). Even at high cross-linker concentrations corresponding to the equimolar ratio of DE to amino groups of chitosan, gelation time was above 1 h for all the studied DEs. Although one can expect that the probability of cross-links formation increases along with the increasing DE chain length, a non-linear dependence on the longest gelation time in the BDDE-chitosan solution was observed at both cross-linking ratios. Most likely, this can be related to the lower water solubility (WS) of BDDE (logWS = −0.183 and 0.47 for BDDE and EGDE, respectively [47,48]) and formation of the microphase, in which self-oligomerization of BDDE can compete with the cross-linking reaction.

Long gelation time is an important advantage of DEs over GA, allowing the fabrication of cryogels in a very broad range of cross-linkers concentrations: colorless chitosan cryogels, which maintain shape of the mold, were obtained at DE:CH molar ratios 1:1–1:20 (Figure 1B).

### 2.2. Cross-Linking Mechanism 

Since DEs can participate in a side reaction with water [31], it is important to understand how the reactivity of DEs in target (cross-linking) and side reactions depends on the cross-linker chain length. Another issue related to the cross-linking mechanism is the completeness of the epoxide ring-opening reaction, which is correlated to potential cytotoxicity of the materials. To addressed these points, FT-IR spectra of chitosan hydrogels and cryogels cross-linked with EGDE, BDDE, and PEGDE at an equimolar ratio were recorded (Figure 1C–E). In the wavenumber range 1200–800 cm^−1^ one can distinguish bands corresponding to C–O–C bridges in DEs (1092–1099 cm^−1^) and chitosan (asymmetric stretching of the C–O–C bridge at 1152 cm^−1^ and C–O stretching at 1064 cm^−1^ and 1024 cm^−1^ [49,50]). Changes in the intensity and position of these bands in spectra of hydrogels and cryogels in comparison with those of the reagents reflect semi-quantitatively the degree of chitosan modification with DEs. Unreacted epoxide groups can be monitored in the wavenumber range 901–910 cm^−1^, where their bands can be distinguished from the bands at 945 and 895 cm^−1^ corresponding to the symmetric vibrations of the C–O–C bonds [51].

**Scheme 1:**
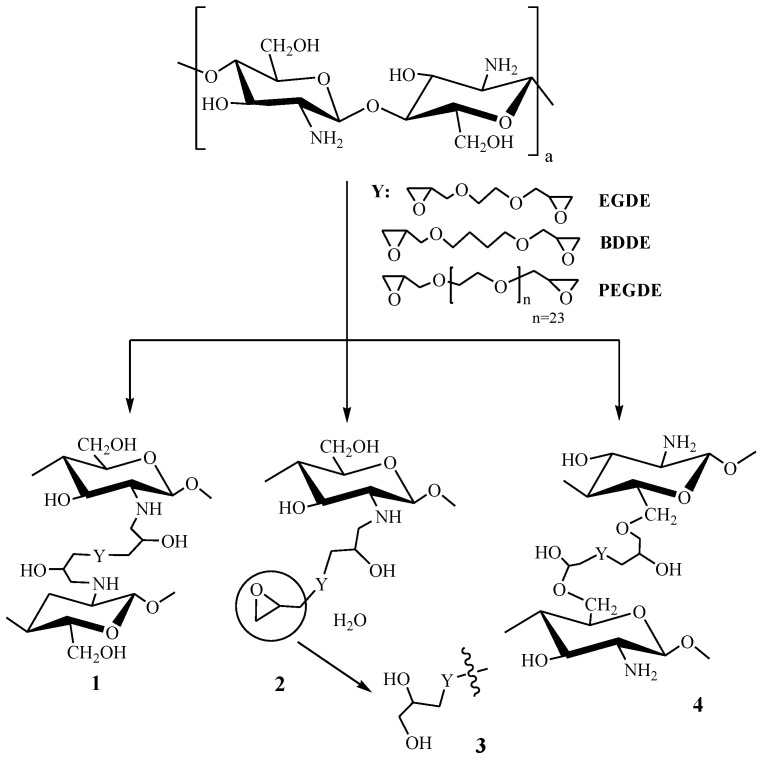
Scheme of possible reactions in chitosan-DE solutions.

**Figure 2:**
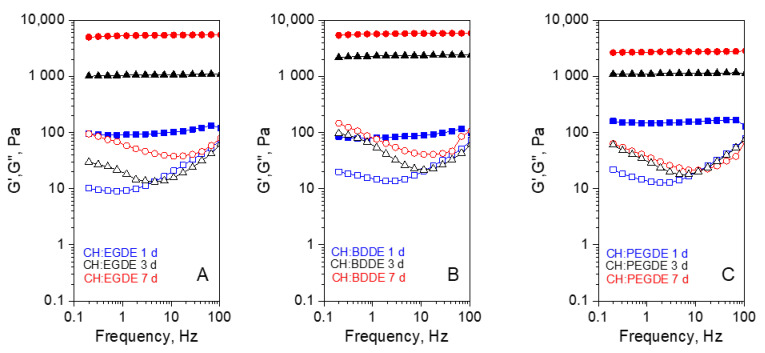
Frequency sweep tests in 3% chitosan solutions with equimolar concentration of the cross-linker at 1st (1 d), 3rd (3 d) and 7th (7 d) day of gelation: cross-linking with EGDE (**A**), BDDE (**B**), and PEGDE (**C**). Open symbols—loss moduli; closed symbols—storage moduli.

First of all, we have analyzed the presence of unreacted epoxides in reaction mixtures at 1st, 3rd, and 7th day of gelation at room temperature (Figure 1C–E and Figure S1, Supplementary Materials). If we assume that the cross-linking reaction with bifunctional DE proceeds via chitosan amino groups (Scheme 1, structure 1), at an equimolar ratio, the unreacted epoxide groups are expected to be found in the reaction mixtures at all time points. However, by the 7th day of gelation, unreacted epoxide groups were detected only in reaction mixtures of chitosan with BDDE and PEGDE (Figure 1D,E). When EGDE was used as a cross-linker, already by the 3rd day of gelation, the intensity of the band corresponding to the epoxide ring remarkably decreased, and the band shifted to the shorter wavenumbers (Figure S1A, Supplementary Materials), where it overlapped with symmetric vibrations of the C–O–C bond in chitosan at 895 cm^−1^. Since the storage modulus of the EGDE-cross-linked hydrogel, as well as of other hydrogels, gradually increased during 7 days (Figure 2), one can conclude that EGDE was simultaneously consumed in both target (cross-linking, Scheme 1, structure 1) and side reactions (grafting and epoxy group hydrolysis, Scheme 1, structures 2 and 3). Hydrolysis of DEs can proceed in aqueous medium at room temperature [52].

To further discern the reaction pathways between grafting without cross-linking and loss of reagent due to epoxy group hydrolysis (Scheme 1, structures 2 and 3), we have analyzed the FT-IR spectra of hydrogels on the 7th day of gelation before and after removal of unreacted chemicals. Hydrogels washed with water/ethanol solution are labeled as EtOH in Figure 1C–E. In the wavenumber range 1200–1000 cm^−1^, most notable changes after removal of the unreacted cross-linker were observed for the CH:PEGDE hydrogel, in which the weight % of chitosan in the reaction mixture at an equimolar ratio was minimal (24.6%). After removal of the unreacted cross-linker on the 7th day of gelation, the most intensive band with a maximum at 1085 cm^−1^, which results from overlapping of PEGDE C–O–C band (1096 cm^−1^) and chitosan C–O stretching (1064 cm^−1^), shifted to the shorter wavenumbers (band maximum at 1073 cm^−1^)—Figure 1E. The latter correlates to the increased content of chitosan in the product in comparison with the reaction mixture, i.e., it proves presence of free unreacted PEGDE after 7 days of gelation at an equimolar PEGDE:CH ratio. Changes in the FT-IR spectra after removal of unreacted chemicals from hydrogels cross-linked with BDDE and EGDE were less obvious (Figure 2A,B) due to approximately twice lower weight % of chitosan in reaction mixtures. Unreacted epoxy groups, which could potentially induce cytotoxicity of the cross-linked materials, have not been identified in the washed hydrogels, although they were detected on the 7th day in reaction mixtures containing BDDE and PEGDE.

Finally, we can emphasize the following important differences in the FT-IR spectra of chitosan cryogels in comparison with hydrogels (both after removal of the unreacted chemicals): (i) maxima of the overlapped bands corresponding to C–O–C in DEs and C–O bonds in chitosan shifted in cryogels to the lower wavenumbers, indicating the decrease in the cross-linker contents in the reaction products, i.e., a drop of reactivity at subzero temperature; (ii) decrease in the cross-linker content in cryogel over hydrogel was most pronounced in case of PEGDE indicating its lower overall reactivity at subzero temperature; (iii) a shoulder at 1152 cm^−1^ and a band at 895 cm^−1^ corresponding to the stretching and symmetric vibrations of the C–O–C bridge in chitosan were clearly seen in the spectrum of CH:EGDE cryogel, suggesting the highest weight % of chitosan in this material. Although, at the same cross-linking density, the weight % of chitosan in cryogels cross-linked with EGDE and BDDE is expected to differ insignificantly, the FT-IR spectrum of CH:BDDE cryogel was much more similar to that of CH:PEGDE. In other words, % of the cross-linker in cryogels CH:BDDE can be higher than expected from the stoichiometric molar ratio of DE to NH_2_ group of chitosan. One should mention that, despite lower reactivity of DEs at subzero temperature, the unreacted epoxy groups were not identified in FT-IR spectra of cryogels (Figure 1C–E), whereas high functional activity (the high proportion of active cells and low proportion of apoptotic and dead cells) of human primary human fibroblasts cultured for 10 days in chitosan cryogels cross-linked with BDDE and PEGDE was confirmed (Figure S2, Supplementary Materials).

Since the cross-linking mechanism and the type and number of cross-links strongly affect mechanical properties and degradability of hydrogels and cryogels [14,53], more information on chemical composition of the fabricated cryogels were obtained from the CHN analysis. The degrees of chitosan modification (MD) with all three cross-linkers were calculated for cryogels obtained at several DE:CH molar ratios (Figure 1F). The most important conclusion from these data is concerned with the highest MD values for BDDE-cross-linked cryogels. The MD value for BDDE at an equimolar ratio of reagents significantly exceeded value expected from stoichiometric interaction via chitosan amino groups. The latter finding confirms our preliminary conclusion derived from FT-IR spectroscopy data that the content of chitosan in hydrogel and cryogel cross-linked with BDDE is lower than in EGDE-cross-linked materials. Although one cannot exclude the possibility of DEs reaction with chitosan via hydroxy groups (Scheme 1, structure 4), hydroxy groups of polysaccharides are usually reactive in alkaline media [45].

The most reasonable explanation of high MD value for BDDE-cross-linked cryogels is related to the formation of the block copolymer networks due to BDDE oligomerization, as it was hypothesized in the Section 2.1 considering the longest gelation time in the presence of this cross-linker. Possible mechanism of BDDE oligomerization can be explained as follows: when hydrolysis of the second epoxy group in the graft (Scheme 1, structure 2) proceeds faster than cross-linking, the terminal alcohol group (Scheme 1, structure 3) can further interact with another molecule of BDDE. The possibility of such oligomerization reactions between amines and glycidyl ethers was reported in [54]. Higher probability of this reaction in case of BDDE can be related to the difference in side reaction kinetics depending on the type of the cross-linker, which is beyond the scope of our investigation.

It can be also mentioned that, despite lower reactivity at subzero temperature, the probability of cross-links formation in a partially frozen system often increases in comparison with gelation at room temperature due to concentration of the reagents in the unfrozen microphase, the so called cryoconcentration effect [55]. This allowed fabrication of mechanically stable chitosan cryogels from hydrochloric acid solutions at much lower concentrations of DEs than it was earlier reported for cross-linking in alkaline media [31] and in acetic acid solutions [33] at room or elevated temperature.

### 2.3. Morphology, Swelling, and Permeability of Chitosan Cryogels

Although cytotropic gelation is one of the most efficient approaches to fabricate porous materials with high permeability, examples of chitosan-based monolith cryogels with good flow-through performance are rather limited [24,38,46,56,57,58,59] in comparison with numerous reports on chitosan cryogels in general. Theoretically, hydrodynamic properties of cryogels vary with the pore structure [57] and the density of cross-links, which affects the degree of the polymer swelling and, thus, the hydrated pore size [23].

Permeability of the earlier reported GA-cross-linked chitosan cryogels increased along with the increase in cryogelation temperature, i.e., the formation of larger pores [60], and with the cross-linking density, leading to the lower swelling of pore walls [46]. When the pore diameter in chitosan cryogels was increased from 60 to 140 µm through the fine tuning of ice crystal growth conditions, the 2.5-fold increase in permeability was observed without changes in swelling [57]. Negative correlation between swelling and permeability of GA-cross-linked chitosan cryogels [46] did not hold in general for DE-cross-linked cryogels. In contrast, permeability increased along with the decrease in the DE concentration (Figure 2A,B and Figure S3, Supplementary Materials). This discrepancy could result from the difference in morphology (pore size) and swelling of the cryogels in dependence on the type of the cross-linker.

Unlike GA, DEs can be considered as dual-purpose cross-linkers with the function of plasticizing agents, whose hydration depends on the glycol chain length [61]. Thus, the increase in the DE concentration can have an opposite effect on swelling of the polymer network due to hydration of the cross-links. Earlier we demonstrated that pore walls of PEGDE-cross-linked chitosan cryogel were thicker than walls in EGDE-cross-linked cryogels, which resulted in high flow resistance of the former material [34], while the correlation between the DE chain length and the DE:CH molar ratio and cryogels properties (swelling, pore size, and permeability) was not established.

**Figure 3:**
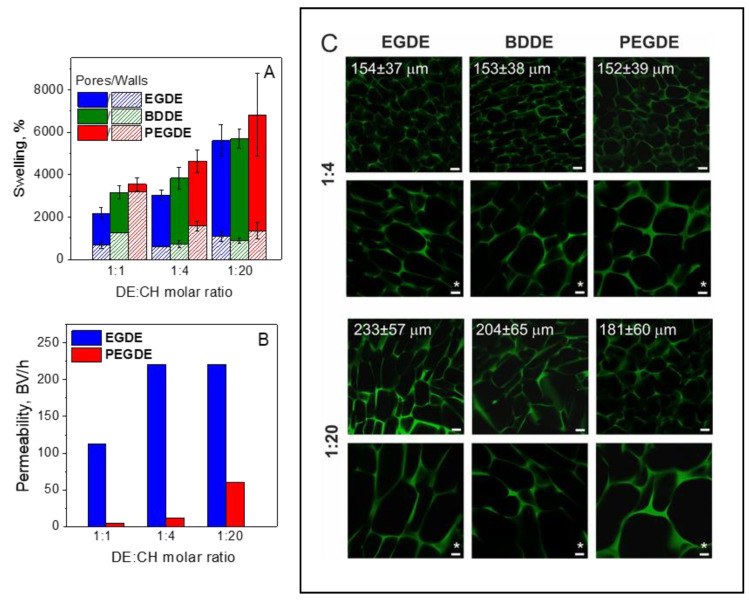
Swelling of chitosan cryogels cross-linked with EGDE, BDDE, and PEGDE at different DE:CH molar ratios (**A**). Dependence of permeability (flow rate, bed volumes (BV)/h) of monolith chitosan cryogels cross-linked with EGDE and PEGDE at different DE:CH molar ratios (**B**). Confocal laser scanning microscopy (CLSM) images and average pore sizes of chitosan cryogels cross-linked with EGDE, BDDE, and PEGDE at DE:CH molar ratios of 1:4 and 1:20, Scale bar—100 µm and 50 µm for images with asterisk * (**C**).

Figure 3A,B show that the DE chain length strongly affected swelling and permeability of DE-cross-linked cryogels. Permeability increased with total swelling, but decreased along with the increase in the polymer phase (walls) swelling, which was maximal for PEGDE-chitosan (Figure 3A). Up to the DE:CH molar ratio of 1:8, both total swelling and swelling of the pore walls increased along with the increase in the DE chain length (Figure S3, Supplementary Materials). Further decrease in the cross-linkers concentration eliminated the difference between cryogels, whereas at the DE:CH molar ratio of 1:20 the swelling mean values for all the materials were not different at *p* = 0.05. Thus, permeability of both GA- and DE-cross-linked cryogels decreased along with the increase in pore walls swelling. However, negative correlation between pore walls swelling and cross-linker concentration found for GA did not hold for DEs, since the decrease in chitosan swelling due to cross-linking was partially compensated by hydration of the glycol cross-links.

Analysis of the pore size, as the second factor affecting cryogels hydrodynamic properties, was performed only for the permeable materials with the lowest and the highest cross-linking density corresponded to the molar ratios of DE:CH 1:20 and 1:4, respectively. Representative CLSM images of the cryogels porous structure are shown in Figure 3C. At high cross-linker concentrations (1:4), the average pore size was close to 150 µm independently of the DE chain length. The increase in permeability of the DE-cross-linked cryogels at low cross-linking densities (molar ratio 1:20), despite high swelling, correlates to the larger pore size of these cryogels, ranging from 181 to 233 µm (Figure 3C).

The pores in DE-cross-linked cryogels were significantly larger than those in GA-chitosan cryogels [5,46,60,62] and lyophilized chitosan scaffolds [63], which in general had dimeters < 100 µm. One of the reasonable explanations of the fact that the pore sizes of GA-cross-linked cryogels were smaller and did not vary greatly with chitosan molecular weight and cryogelation temperature [23,46,60] is related to very fast rate of chitosan gelation in the presence of GA [21,22]. An abrupt increase in viscosity of the freezing solution restricts the growth of ice crystals, and, thus, results in formation of the smaller pores [57]. Gelation of chitosan with DEs was significantly slower than with GA and at low DE concentrations (1:20), gelation time exceeded 24 h (Figure 1A). Therefore, a slow increase in viscosity in freezing solutions did not hinder the solvent crystallization, allowing the formation of larger pores more suitable for flow-through applications and cell culture.

One should mention that, since the viscosity of chitosan solutions strongly depends not only on the concentration and molecular weight of the polymer, but also on pH, ionic strength, anionic form, and deacetylation degree [64,65,66], it must be strictly controlled in the process of cryogel fabrication to obtain reproducible results.

### 2.4. Mechanical Properties 

**Figure 4:**
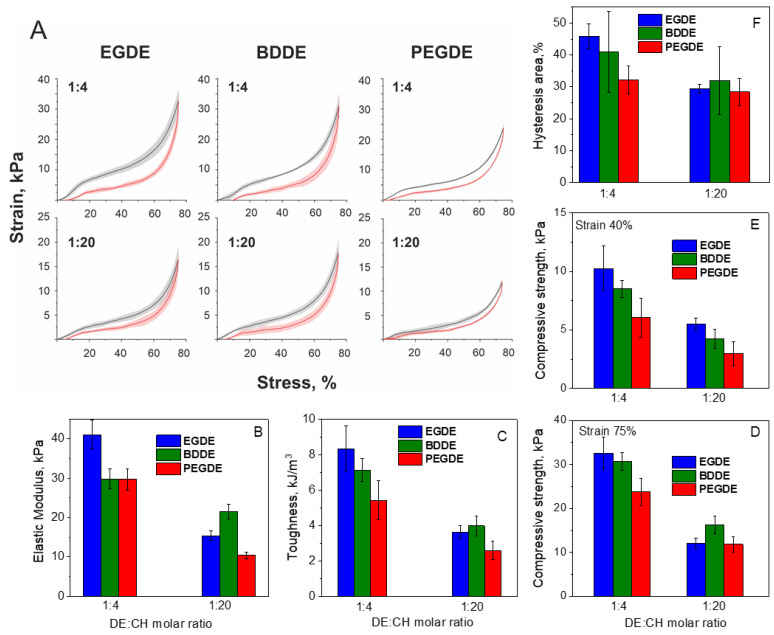
Mechanical properties of the swollen chitosan cryogels cross-linked with EGDE, BDDGE, and PEGDGE at the DE:CH molar ratios of 1:4 and 1:20: strain–stress curves of uniaxial compression (**A**), elastic moduli (**B**), toughness (**C**), compressive strength at 75% (**D**) and 40% (**E**) deformation, and hysteresis (**F**).

Strain–stress curves of chitosan cryogels under uniaxial compression (loading-unloading cycle) have a typical for the biological tissues non-linear shape with a strain-stiffening behavior (Figure 4A). Three regions can be clearly distinguished in all curves [67,68]: (i) linear elastic regime (0–15% strain); (ii) pores closer, where free water is squeezed with a minimal variation in stress (~15–55% strain); (iii) pore walls densification (>55% strain, wall stiffness). Depending on the type of application, different parameters are used to characterize mechanical properties of cryogels [10]. Although bulk and wall stiffness are sometimes calculated from the first and the third regions of the strain–stress curves [69], the choice of the range for linearization is to some extend subjective and material-dependent that makes the comparison ambiguous. The compressive strength at different levels of sample deformation and integral characteristics, such as toughness and hysteresis, are more reliable parameters to elucidate the effect of the cross-linker type and concentration on the mechanical properties of the cryogels.

The highest elasticity, i.e., the ability of the material to retune to its original shape after deformation, was observed for PEGDE-cross-linked cryogels, which recover 98–99% of the original height after unloading and absorption of the squeezed water—Figure 4A. These cryogels were also characterized by the lowest hysteresis, which can be related to the reduction of internal friction due to the weakening of intermolecular hydrogen bonding in chitosan, i.e., plasticizing activity of the hydrated PEG-cross-links. All other cryogels also swelled during unloading with a recovery 90–96% of the height. After reswelling with addition of extra volume of water, shape recovery was close to 100%.

Although the loading-unloading cycles were recorded up to 75% strain, all cryogels could be compressed at least up to 90% strain without cracks propagation and loss of the sample integrity. By varying the DE chain length and concentration, the cryogels with elasticity moduli from 10.4 ± 0.8 to 41 ± 3 kPa (Figure 4B), toughness from 2.68 ± 0.5 to 8.3 ± 0.1 kJ/m^3^ (Figure 4C), and compressive strength at 75% strain from 11 ± 2 to 33 ± 4 kPa (Figure 4D) were fabricated. At high cross-linking ratios (DE:CH 1:4), toughness (Figure 4C), hysteresis (Figure 4F), and compressive strength (Figure 4D,E) of the cryogels decreased along with the increase in the cross-linker chain length demonstrating that elasticity was gained at the expense of strength. The stiffest and toughest in the row EGDE-cross-linked cryogel had a compressive strength (33 ± 4 kPa at 75% deformation) and an elastic modulus (41 ± 3 kPa), which are higher than those reported for the GA-cross-linked chitosan cryogels [62] and hydrogels [70].

At the DE:CH molar ratio of 1:20, the correlation between mechanical properties of cryogels and cross-linker chain length was less explicit, since at low DE concentrations, the gelation time significantly increased (Figure 1A), the fastest deactivation of EGDE epoxy groups in the side reaction with water (Scheme 1, structures 2 and 3, Figure 1C) can be responsible for lower cross-linking efficiency. This can explain lower stiffness, compressive strength, and toughness of EGDE- over BDDE-cross-linked cryogel at this DE:CH ratio, although at high cross-linking densities, EGDE yielded the stiffest and toughest cryogel. One should also mention that BDDE-cross-linked cryogels were characterized by the highest standard deviations for the hysteresis and higher probability of cracks at high loading. We assume that the latter can result from the less regular structure of the polymeric network due to the formation of microphases and oligomerized grafts with different length.

Pearson correlation analysis showed (Table S1, Supplementary Materials) that the compressive strength of chitosan cryogels decreased with increase in the pore size and the cross-linker chain length, while the strongest negative correlation was observed with cross-linker concentration, i.e. DE:CH molar ratio (r = −0.85), and total swelling (r = −0.89).

### 2.5. Enzymatic Hydrolysis

**Figure 5:**
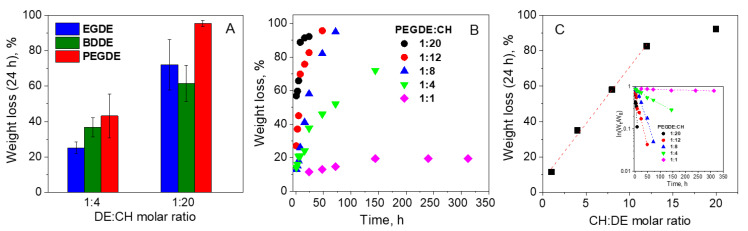
In vitro degradation of chitosan cryogels with β-glucanase, 330 U/mL, pH 7.4, T = 37 °C: weight loss after 24 h for cryogels cross-linked with EGDE, BDDE, and PEGDE at the DE:CH molar ratios of 1:4 and 1:20 (**A**); kinetics of PEGDE-cross-linked cryogel degradation (**B**); and weight loss after 24 h (**C**) depending on PEGDE:CH or CH:PEGDE molar ratio; linearization of kinetic curves using pseudo-first-order kinetics equation (insert in (**C**)).

Susceptibility of chitosan cryogels to enzymatic hydrolysis is an important factor affecting the performance and fate of chitosan scaffolds in vivo [71] and drug release rate from loaded degradable hydrogel films [72]. Chitosan with varying degrees of acetylation and substitution are susceptible to a variety of specific and non-specific enzymes [40] including endogalactosaminidase, which is present in human serum and leukocytes and demonstrates the chitinase activity [73,74]. To elucidate how the suggested here approach to chitosan scaffolds fabrication can be used to tune the enzymatic degradation rate, we first investigated the effect of the cross-linker chain length on the weight loss of highly cross-linked (DE:CH of 1:4) scaffolds in the presence of β-glucanase isolated from *Myceliophthora fergusii* (Figure 5A). According to the work [75], this enzymatic system shows chitinase and chitosanase activity and performs endocleavage of glycosidic bonds of the polymer chain.

Figure 5A shows that, at high cross-linker concentrations, the weight loss after 24 h increased along with the increase in the cross-linker chain length in the order EGDE < BDDE < PEGDE with maximal and minimal degradations of 57 ± 12% and 29 ± 3% for PEGDE:CH and EGDE:CH, respectively. One should mention that degradation of the BDDE:CH cryogel was remarkably lower (37 ± 5%) in comparison with the chitosan film cross-linked in isopropanol at 45 °C at much higher BDDE concentrations [31]. At low cross-linking densities, the weight loss of chitosan cryogels was in average twice higher and reached 95 ± 2% for PEGDE:CH. A non-linear dependence of degradation on the DE chain length for weakly cross-linked cryogels is similar to that observed for mechanical properties and can be related to faster deactivation of EGDE in side reactions, i.e., lower cross-linking efficiency.

To find a correlation between the susceptibility to enzymatic degradation, cross-linking conditions, and cryogel characteristics, Pearson correlation coefficients were determined for the following parameters: cross-linker chain length, molar ratio DE:CH (1:4/1:20), pore size, total swelling, polymer swelling, and compressive strength at 75% strain (Table S1, Supplementary Materials). The weight loss after 24 h was found to be affected by all of these parameters, but was highly correlated to the CH:DE molar ratio (r = 0.77), hysteresis (r = −0.74), total swelling (r = 0.84), and compressive strength (r = −0.85).

To sum up, the most significant weight loss was found for the most elastic PEGDE-cross-linked cryogels with the highest swelling and lowest toughness and compressive strength. To tune the enzymatic degradability of chitosan cryogels in the broad range, the PEGDE:CH ratio was varied from 1:1 to 1:20, and the degradation kinetics of these materials was investigated. The weight loss of PEGDE:CH cryogels after 24 h in β-glucanase solution varied in the range 11–95% (Figure 5B) and decreased linearly along with the increase in the cross-linking density in the range of PEGDE:CH molar ratios 1:1–1:12 (Figure 5C). This dependence can be correlated to the involvement of amino groups to the cross-linking reaction and, as a result, the reduction in the number of structural fragments susceptible for the enzymatic attack. Most likely, that fast and deep degradation of BDDE- and PEGDE-cross-linked chitosan films reported earlier results from an inefficient cross-linking under heterogeneous conditions in methanol [31] or in acetic acid solution [43], respectively.

The enzymatic degradation of PEGDE-cross-linked chitosan cryogels followed the first-order degradation kinetics (Figure 5C, insert) with the degradation rates in the range 0.00033–0.20502 h^−1^ (Table 1). With the 612-fold decrease in the degradation rate at the 20-fold increase in the cross-linker concentration, susceptibility of chitosan cryogel to enzymatic hydrolysis was the parameter most sensitive to the variation of cross-linking conditions.

## 3. Conclusions

Diglycidyl ethers of glycols (DEs) with dual functionality of the cross-linkers and plasticizing agents can be considered as a more powerful alternative to glutaraldehyde (GA) for fabrication of chitosan hydrogels and cryogels, but their application is significantly limited by slow progress in chemistry of epoxide–amine reactions in acidic chitosan solutions.

This fact has motivated us to verify whether the variation of DE chain length can be used as an efficient tool to tune swelling, mechanical properties, and enzymatic degradability of covalently cross-linked chitosan cryogels, since all these characteristics are strongly correlated to the density of the pore walls.

We have shown that one of the important advantages of DEs over GA consists of slower cross-linking, which does not interfere with solvent crystallization and facilitates formation of larger pores, i.e., more permeable cryogels. At low cross-linking densities, the average pore diameter increased with decreased DE chain length and varied in the range 181–233 µm.

At high cross-linking ratios (DE:CH 1:4), the toughness, hysteresis, and compressive strength of the cryogels decreased along with the increase in the cross-linker chain length, demonstrating that elasticity was gained at the expense of strength. The stiffest and toughest in the row EGDE-cross-linked cryogel had a compressive strength (33 ± 4 kPa at 75% deformation) and an elastic modulus (41 ± 3 kPa) higher than those reported for the GA-cross-linked chitosan cryogels and hydrogels [67,68,69,70].

The swelling increased in the order of cross-linkers: EGDE < BDDE < PEGDE. Hydration of the DE cross-links weakens intermolecular hydrogen bonding in chitosan and, therefore, facilitates enzymes penetration to the polymer network. This resulted in increased weight loss of chitosan cryogels in the presence of β-glucanase with increased DE chain length. For the most soft and elastic PEGDE-cross-linked cryogel (DE:CH 1:20), weight loss after 24 h reached 95%, while the degradation rate constant decreased 612-fold at a 20-fold increase in the PEGDE concentration from DE:CH 1:20 to 1:1. This confirms that susceptibility to enzymatic hydrolysis can be used as a parameter very sensitive to cross-linking conditions and the resulting density of the pore walls that is important for understanding cell–scaffold interactions and interpretation of in vivo biodegradability data.

## 4. Materials and Methods

### 4.1. Materials 

Chitosan was purchased from BioLog Heppe GmbH (Landsberg, Germany). The degree of acetylation (DA) determined by ^1^H NMR spectroscopy was 0.9, and the viscosity-average molecular weight was 30 kDa. Cross-linking agents—poly(ethylene glycol) diglycidyl ether, average Mn 500 (PEGDE), and 1,4-butanediol diglycidyl ether (BDDE)—were purchased from Sigma-Aldrich (St. Louis, MO, USA). Ethylene glycol diglycidyl ether (EGDE) was purchased from J&K Scientific (Guangzhou, China). Other reagents were of analytical grade.

### 4.2. Methods

#### 4.2.1. Determination of Gelation Time 

The 3% chitosan solution was prepared by dissolution of the polymer powder in hydrochloric acid at a stoichiometric ratio to amino groups, and the pH was adjusted to 5.5 using 0.1 M NaOH solution. Precalculated amounts of EGDE, BDDE, PEGDE corresponding to the DE:CH (chitosan) molar ratios of 1:1 and 1:20 were added under stirring to the chitosan solutions at room temperature (25 °C), after mixing for 15 min and left for the gelation. The frequency sweeps curves were recorded in chitosan solutions after cross-linkers addition in the range from 0.2 to 100 Hz at a temperature of 25 °C and a constant strain of 5% using a Physica MCR 301 rheometer (Anton Paar GmbH, Graz, Austria) with a plate–plate measuring system of a diameter of 25 mm. Gelation times were determined from the crossover points of the time dependencies of storage (G′) and loss (G″) moduli at a frequency of 11.2 Hz.

#### 4.2.2. Cryogel Fabrication 

The 3% chitosan solutions prepared as described in Section 4.2.1 with a viscosity of 0.63 ± 0.04 Pa·s at 11.2 Hz were used for cryogel fabrication. Precalculated amounts of EGDE, BDDE, and PEGDEGE corresponding to the molar ratios to chitosan amino group from 1:1 to 1:20 were added under constant stirring to the chitosan solutions at 25 °C mixed for 5 min and placed into the plastic syringes (inner diameter of ~1 cm) and kept in a freezer at −10 °C for 12 days. After thawing at 25 °C, the cryogels were washed inside syringes using peristaltic pump to feed at least 200 mL of distilled water through the material to remove unreacted chemicals.

#### 4.2.3. Elemental Analysis and FT-IR Spectroscopy 

Elemental compositions (C, H, N) of cryogels were determined in triplicates using an EuroEA3000 CHNS analyzer (Eurovector, Pavia, Italy). The degree of chitosan modification (MD) with the cross-linking agent was calculated using the following formula:

(1) D=(CNcryogel−CNchit)/n

where C/N_cryogel_ and C/N_chit_ are the atomic ratios carbon/nitrogen in cryogels and original chitosan, respectively; n is the number of carbon atoms in the cross-linking agent.

Fourier transform infrared (FT-IR) absorbance spectra of hydrogels (reaction mixtures) and cryogels were recorded using an IR Affinity-1 spectrometer with a QATR 10 single-reflection ATR accessory (Shimadzu, Kyoto, Japan) in the range 400–4000 cm^−1^. The number of scans was 32, the resolution was 16, and the Happ–Genzel apodization function was used for spectra processing. The FT-IR spectra were recorded for lyophilized hydrogels (reaction mixtures without purification) obtained as described in Section 4.2.1 after 1, 3, and 7 days of gelation at DE:CH molar ratio 1:1 (samples are labeled as CH:EGDE/BDDE/PEGDE 1 d/3 d/7 d). Additionally, FT-IR spectra were recorded for hydrogels obtained after 7 days of gelation and sequentially washed overnight with water and water/ethanol mixture (20:80 *v*/*v*) to remove unreacted chemicals (samples are labeled as CH:EGDE/BDDE/PEGDE (EtOH)). The hydrogel:solvent volume ratio was 1:10.

#### 4.2.4. Swelling and Morphology 

Swelling (total swelling) of the cryogels was determined from the difference in weights of swollen and dry material (the measurements were performed for freshly prepared cryogels from wet to dry state). To determine the contribution of free-flowing water in macropores to the total swelling, the swollen cryogels were first squeezed by fingers and weighted, then dried to the constant weight at 90 °C. Swelling of the polymeric phase (walls) was calculated as a difference between weights of squeezed and dried material.

Morphology of the swollen (never dried) chitosan cryogels stained with fluorescein was investigated using a Carl Zeiss LSM 800 confocal laser scanning microscope (Jena, Germany) with regular 10× and 20× objective lens. The excitation and emission wavelengths were set at 493 and at 517, respectively. Images were generated by optical sectioning in the xy planes along the z-axis with 30 optical sections with 2.5 μm intervals. The optical-section series were projected as single images and exported as TIFF files. The pore size distributions were calculated using the ImageJ software V 1.8.0 [76]. The middle part of the syringes were taken for analysis to avoid effects of difference in supercooling conditions, which often results in heterogeneity of the porous structure in cryogel sections from top, middle, and bottom part of the mold [77].

#### 4.2.5. Mechanical Properties

Uniaxial compression tests at a constant speed of 0.01 mm/s were performed for the swollen cylindrically shaped cryogels with a diameter of 10–15 mm and height of 8–10 mm using Physica MCR 301 rheometer (Anton Paar GmbH, Graz, Austria). Loading and unloading strain–stress curves have been calculated from the measured normal force (F_N_) and gap. The elastic modulus (E) was calculated from the linear region (5–15% strain) of the loading curve:

(2) E=l0·FNS·∆l

where F_N_ is the normal force (N), l_0_ is the initial sample height (m), Δl is the change in the sample height (m), and S is the area of the material (m^2^).

Compressive strength at 40 and 75% strain was calculated from the stress–strain curves. Toughness was calculated by integrating the area under the stress–strain curve for the sample deformation up to 75%. The dissipated energy was calculated as an area of the loop between the loading and unloading curves. The hysteresis area was calculated as the percentage ratio between dissipated energy and toughness within one loading cycle.

#### 4.2.6. Enzymatic Hydrolysis

Food-grade enzymatic system (Limited Liability Company “Biopreparat”, Voronezh, Russia) with *β*-glucanase activity of 10,000 U/mL was obtained from *Myceliophthora fergusii*, purified and concentrated. Cryogel degradation was estimated by the gravimetric method as follows: the swollen cryogels were cut to the discs (3 replicates) with diameter of ~10 mm and height ~4 mm (dry weight ~5 mg), squeezed to remove excess of water and placed into vials with 1 mL of Dulbecco’s modified Eagle’s medium (DMEM, #12800017, Gibco™, Thermo Fisher Scientific, Altrincham, UK) containing 330 U/mL of *β*-glucanase. This activity of enzyme was selected from preliminary experiments, which showed 90% drop in viscosity of 4% chitosan solution during 30 min at pH 5 and 330 U/mL of *β*-glucanase. Cryogel disks immersed in enzyme-free DMEM medium were used as a control. Sealed vials with control and test samples were thermostated at 37 °C for 24 h, then cryogels were removed, thoroughly washed with distilled water, and dried at 90 °C to the constant weight. When the degradation kinetics were investigated, discs were removed in predefined intervals of time (t).

The weight loss of the cryogel after immersion time (t) was calculated using the following formula:

(3) $$Weightloss=(\left(W_{0}-W_{t}\right){/W}_{0})\times 100\%$$

where W_0_ is initial weight of the cryogel, W_t_ is the residual weight of the cryogel after the immersion time (t). Calculations were performed for the dry weights of the samples using correlation coefficients accounting for the swelling.

Cryogel degradation kinetics was fitted using pseudo-first-order decay kinetics equation:

(4) $$W_{t}/W_{0}=e^{-\mathrm{kt}}$$

where W_0_ is the initial weight of the cryogel, W_t_ is the residual weight of the cryogel after immersion time (t), and k is the degradation rate constant.

#### 4.2.7. Cryogel Cytotoxicity

Cryogel disks (diameter of 8–10 mm, thickness of 4 mm) were placed in wells of a 24-well culture plate (TPP, Trasadingen, Switzerland), and the primary human dermal fibroblasts were seeded at a density 100 × 10^3^ cells/disk in 1 mL of Dulbecco’s modified Eagle’s medium (DMEM, #12800017, Gibco™, Thermo Fisher Scientific, Altrincham, UK) supplemented with 10% (*v*/*v*) fetal bovine serum (FBS, HyClone, Logan, UT, USA), 3.7 mg/mL sodium bicarbonate (Sigma-Aldrich, St. Louis, MO, USA), 1× mixture of non-essential amino acids (MEM NEAA, Gibco, Waltham, MA, USA), 100 U/mL penicillin (Gibco), and 100 µg/mL streptomycin (Gibco). The samples were cultivated at +37 °C, 5% CO_2_, and 90% relative humidity for 10 days. The medium has been replaced each 2 days. Viability of cells was estimated using flow cytometry after staining cells with 2′,7′-dichlorodihydrofluorescein diacetate (H_2_DCFDA) (Sigma-Aldrich, St. Louis, MO, USA) to assess the mitochondrial activity, TO-PRO-3™ (Invitrogen, Waltham, MA, USA) to detect apoptotic cells, and DAPI (GERBU Biotechnik GmbH, Heidelberg, Germany) to stain dead cells as described in details in [78].

## Figures and Tables

**Table 1:** Rate constants for chitosan cryogel degradation with β-glucanase, pH 7.4, T = 37 °C.

PEGDE:CH Molar Ratio	k_1_ (1/h)	R^2^
1:1	0.00033	0.784
1:4	0.00781	0.99
1:8	0.03964	0.99
1:12	0.05824	0.97
1:20	0.20502	0.88

## Data Availability

Data are available upon request.

## Supplementary Materials

The following supporting information can be downloaded at: https://www.mdpi.com/article/10.3390/gels10070483/s1, Figure S1. FT-IR spectra of reaction mixtures (3% chitosan solution + cross-linker at equimolar concentration) at 1st (1 d), 3rd (3 d) and 7th (7 d) day of gelation: cross-linking with EGDE (A), BDDE (B), and PEGDE (C); Figure S2. Functional activity of primary human fibroblasts cultivated for 3, 7, 10, and 14 days in chitosan cryogel cross-linked with BDDGE (BDDE: CH molar ratio of 1:4) and PEGDGE (PEGDE: CH molar ratio of 1:20). The cells were stained with H_2_DCFDA to assess the mitochondrial activity, TO-PRO-3™ to detect apoptotic cells, and DAPI to stain dead cells followed by flow cytometrical analysis. The data are presented as a mean of three independent experiments. Standard deviations did not exceed 5%; Figure S3. Swelling and permeability (flow rate, bed volumes (BV)/h) of monolith chitosan cryogels cross-linked with EGDE and PEGDE at different DE:CH molar ratios; Table S1. Correlation Matrix of Pearson pairwise correlation coefficients between variables used in the empirical analysis of the effect of fabrication conditions on properties of chitosan cryogels cross-linked with diglycidyl ethers of glycols with different chain length.
