# Supplementary material for: Tuning Mechanical Properties, Swelling, and Enzymatic Degradation of Chitosan Cryogels Using Diglycidyl Ethers of Glycols with Different Chain Length as Cross-Linkers
Source: Gels. 2024 Jul 21;10(7):483. doi: 10.3390/gels10070483 (PMC11276332; doi:10.3390/gels10070483)
Supplement: Supplementary file 1 [file gels-10-00483-s001.zip › gels-3098935-supplementary.pdf]

Supplementary Materials

Tuning mechanical properties, swelling and enzymatic degradation of chitosan cryogels using diglycidyl ethers of glycols with different chain length as cross-linkers

Yuliya Privar[1], Anna Skatova[1], Mariya Maiorova[2], Alexey Golikov[1], Andrey Boroda[2], and Svetlana Bratskaya[1,*]

[1] Institute of Chemistry Far Eastern Branch of the Russian Academy of Sciences, 159, prosp.100-letiya Vladivostoka, 690022 Vladivostok, Russia; sbratska@ich.dvo.ru (S.B.)
[2] A.V. Zhirmunsky National Scientific Center of Marine Biology, Far Eastern Branch of Russian Academy of Sciences, 17, Palchevskogo street, 690041 Vladivostok, Russia; borodandy@gmail.com (A.B.)
* Correspondence: sbratska@ich.dvo.ru

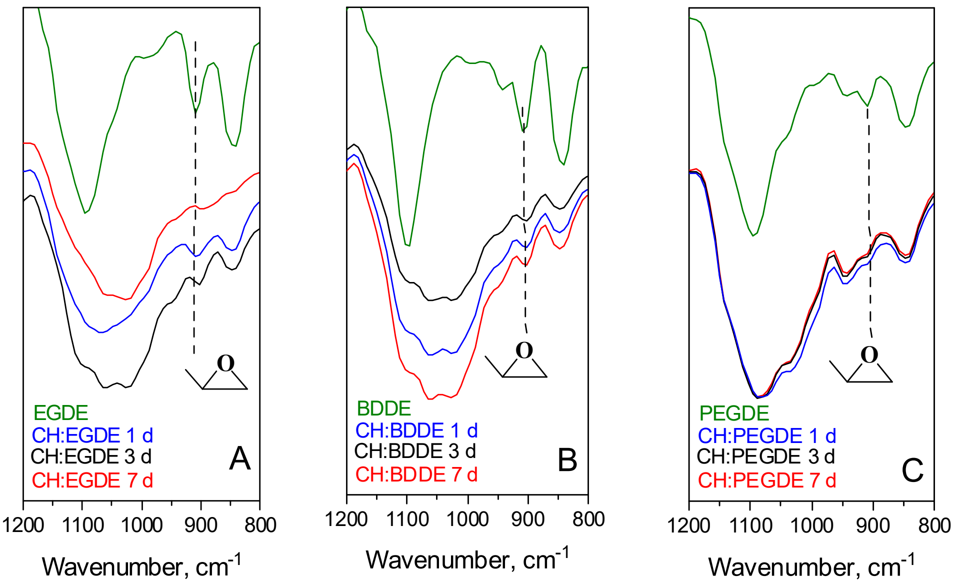

Figure S1. FT-IR spectra of reaction mixtures (3% chitosan solution + cross-linker at equimolar concentration) at 1st (1d), 3rd (3d) and 7th (7d) day of gelation: cross-linking with EGDE (A), BDDE (B), and PEGDE (C).

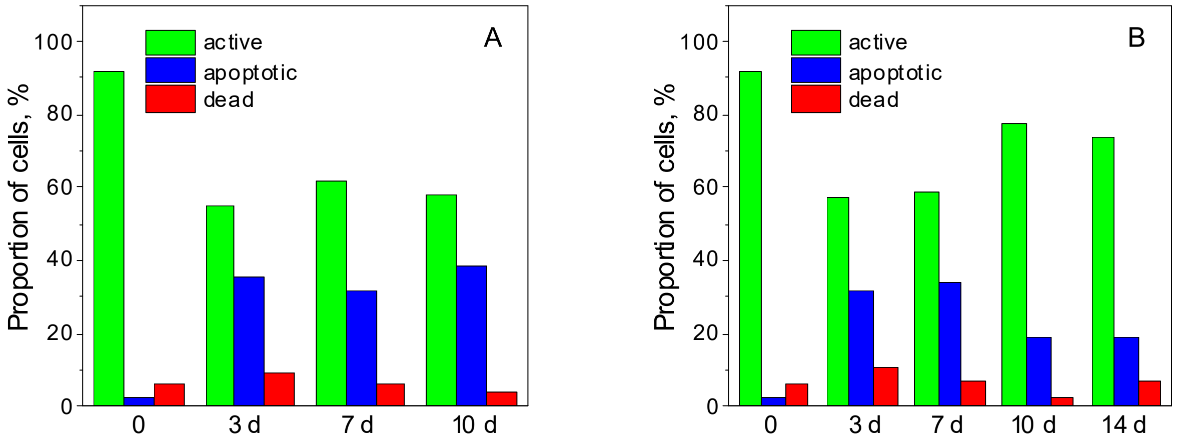

Figure S2. Functional activity of primary human fibroblasts cultivated for 3, 7, 10, and 14 days in chitosan cryogel cross-linked with BDDGE (BDDE: CH molar ratio of 1:4) -A and PEGDGE (PEGDE: CH molar ratio of 1:20) - B. The cells were stained with H_2DCFDA to assess the mitochondrial activity, TO-PRO-3™ to detect apoptotic cells, and DAPI to stain dead cells followed by flow cytometrical analysis. The data is presented as a mean of three independent experiments. Standard deviations did not exceed 5%.

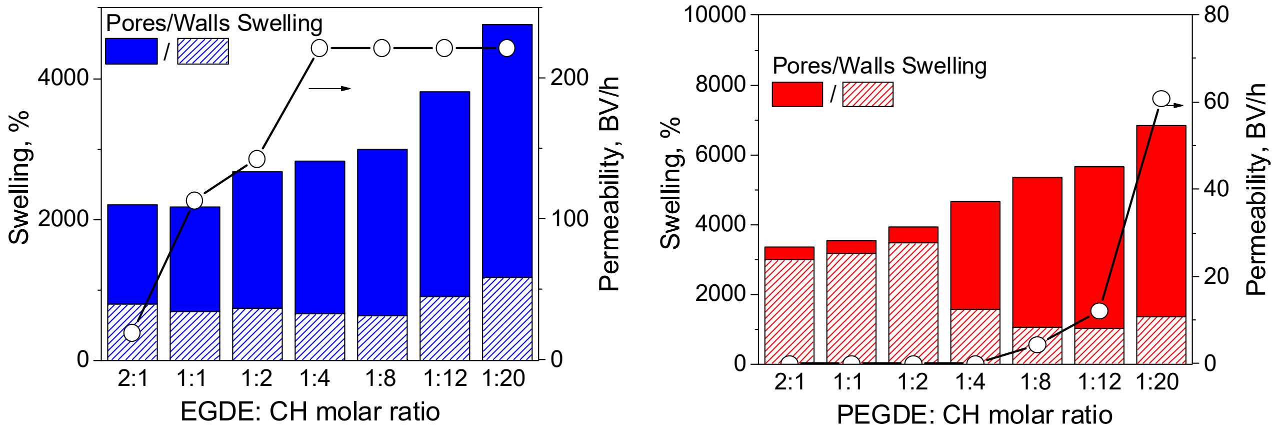

Figure S3. Swelling and permeability (flow rate, bed volumes (BV)/h) of monolith chitosan cryogels cross-linked with EGDE and PEGDE at different DE:CH molar ratios.

Table S1. Correlation Matrix of Pearson pairwise correlation coefficients between variables* used in the empirical analysis of the effect of fabrication conditions on properties of chitosan cryogels cross-linked with diglycidyl ethers of glycols with different chain length

	N_C	CH:DE	Pore size	ST	SW	EnzD	Hys	E-75
N_C	1	0.034	-0.253	0.278	0.737	0.538	-0.428	-0.406
CH:DE	0.034	1	0.892	0.870	0.262	0.767	-0.583	-0.853
Pore size	-0.253	0.892	1	0.779	0.146	0.570	-0.479	-0.698
ST	0.278	0.870	0.779	1	0.593	0.844	-0.677	-0.899
SW	0.737	0.262	0.1469	0.593	1	0.640	-0.541	-0.534
EnzD	0.538	0.767	0.570	0.847	0.639	1	-0.747	-0.853
Hys	-0.428	-0.583	-0.479	-0.677	-0.541	-0.747	1	0.755
E-75	-0.406	-0.853	-0.698	-0.899	-0.534	-0.853	0.755	1
Emax-40	-0.373	-0.836	-0.681	-0.882	-0.489	-0.815	0.786	0.985

*N_C (Number of carbon atoms in cross-linker, N=8, 10 and 23 for EGDE, BDDE, and PEGDE, respectively); CH:DE (Molar ratio CH:DE, 4 – high cross-linking density, 20 – low cross-linking density); ST (Total swelling); SW (Swelling of the pore walls); EnzD (Enzymatic degradability= weigh loss after 24 h); Hys (Hysteresis); E-75 (Compressive strength at 75% deformation)
